# Health impact of probiotics - vision and opportunities

**DOI:** 10.1186/1757-4749-4-1

**Published:** 2012-03-12

**Authors:** Neerja Hajela, G Balakrish Nair, Philip Abraham, Nirmal K Ganguly

**Affiliations:** 1 Yakult India Microbiota and Probiotic Science Foundation, New Delhi, India; 2 Translational Health Science and Technology Institute, Gurgaon, Haryana, India; 3 P.D. Hinduja National Hospital, Mumbai, India; 4 National Institute of Immunology, New Delhi, India

## Abstract

Our understanding of the role of the microbiota in our gut and other sites in our body is rapidly emerging and could lead to many new and innovative approaches for health care. The promise of the potential role of probiotics for the prevention and treatment of enteric and other infections as an effective solution needs to be realized. The meeting report summarizes the insights and learning from a recent symposium, "Health Impact of Probiotics - Vision and Opportunities" conducted in Mumbai by the Yakult India Microbiota and Probiotic Science Foundation and P.D. Hinduja National Hospital, Mumbai. The symposium reflected its objective of unraveling the potential role of probiotics for health benefits through presentations and discussions. Experts clearly highlighted the role of probiotics in improving various aspects of health and in immune modulation. The report also captures the debate and discussions on the challenges that are likely to be encountered for the use of probiotics in the country.

## Introduction

The recent advent of high throughput sequencing technologies has provided new insights into the composition and metabolic activities of the intestinal biome. Today it is recognized that this complex ecosystem is an assemblage of more than 1000 species of microorganisms that exist in harmony (symbiosis) with the host and exert metabolic activities that virtually parallels an organ within an organ. While most of these activities are beneficial for the host some may be deleterious, the nature of effect, being determined by the composition of the flora. Thus a mutualistic relationship between the beneficial symbionts and commensals is paramount for the maintenance of health and wellbeing with dysbiosis resulting in clinical disease. It follows that the gut flora can be manipulated to enhance the beneficial components which represent a promising strategy for the prevention and management of various infective and non - infective disorders.

The symposium started with the welcome address by Prof. N.K. Ganguly, Distinguished Biotechnology Research Professor, Department of Biotechnology, National Institute of Immunology, New Delhi. He gave a broad introduction to the topic, emphasizing the central role of the gut flora in the improvement of health. He stated that of the many proposed interventions, probiotics offer immense potential for the prevention of a variety of diseases. It is therefore not surprising that the past decade has witnessed tremendous progress in the area of probiotic research with scientific evidence quickly accumulating to validate their possible role as important therapeutic and preventive strategy for gastrointestinal diseases, treatment and prevention of allergic disorders, chronic inflammatory diseases, prevention of cancers and reduction of respiratory diseases.

Dr. R.A. Badwe, Director, Tata Memorial Cancer Hospital, Mumbai, the Chief Guest reemphasized the significant role of the gut flora in maintaining health and stated that although most of the claims and benefits associated with probiotic usage are being established, they mainly represent the findings from the West and therefore probiotics have found greater acceptance in that part of the world. The potential for their use in the developing world has not been adequately investigated. With the realization that infectious and non-infectious diseases account for a large majority of deaths annually in resource limited settings there is optimism about the ability of interventions such as probiotics to prevent health disparities. However, since colonization with a probiotic is dependent on the interplay of multiple factors in the intestinal mileu, inherent differences in gut microbial ecology may significantly impact the functioning of the probiotic strain and therefore requires a thorough understanding and much more evidence needs to be generated in these settings.

### Yakult India Microbiota and Probiotic Science Foundation

Recognizing this need, a group of eminent scientists formed the Yakult India Microbiota and Probiotic Science Foundation. This foundation was registered as a society on 9th November 2011 under the Societies Registration Act XXI of 1860. The Foundation will aim at providing a common scientific platform for basic scientists and clinicians to share and exchange knowledge and views and to expand into newer areas of probiotic research. While the foundation will channelize International knowledge and expertise in the field of probiotics it will also promote collaborative research in the development of probiotics as well as foster and maintain research links with scientists of similar interest. The foundation endeavors to do this through an Annual Probiotic Symposium, which will blend fundamental and applied research related to the use of probiotics for the enhancement of human health. With this in mind, the first symposium of the Foundation with the theme "Health Impact of Probiotics - Vision and Opportunities" was convened on December 10 and 11, 2011 in Mumbai.

This report summarizes the discussions and deliberations that ensued over the two days of the symposium. We hope this will prompt basic scientists, microbiologists and nutritionists to bring new perspectives to the science of probiotics and clarify some of the major issues such as efficacy and acceptable outcomes that surrounds implementation of probiotic usage in the country. The challenge of designing randomized controlled trials for understanding and targeting particular biomarkers and end points for the disease and identifying strain specific health benefits to mark a road map that may be followed to allow for probiotic usage in the country needs to be addressed.

### Unraveling the effects of probiotics in Gastrointestinal (GI) disorders

Evidence for the utility of probiotics in the prevention and treatment of GI disorders in the pediatric population and in adults was presented by Prof. Philip Sherman, Professor of Pediatrics, Microbiology and Dentistry, Research Institute, University for Sick Children, University of Toronto, Canada and Dr. Philip Abraham, Senior Consultant Gastroenterologist and Hepatologist at P.D. Hinduja National Hospital, Mumbai.

Presenting global evidence for their utility in children, Prof. Sherman illustrated that evidence through randomized controlled trails have demonstrated that certain probiotic strains are more effective than placebo in a variety of conditions affecting the gastrointestinal tract. Multiple meta-analyses indicate effectiveness in reducing the duration of acute enteritis in pre-schoolers and in reducing the frequency of necrotizing enterocolitis in pre-term babies [1,2]. There is some evidence supporting the use of selected probiotic agents to either prevent or treat antibiotic-associated diarrhea and *Clostridium difficile *infection. In Prof. Sherman's opinion, more data is, however, necessary to confirm the efficacy of probiotic agents, either solely or in combination, in the management of various chronic inflammatory bowel diseases and in allergic enterocolitis. According to him, in future, probiotic strains are likely to be chosen for use in specific clinical settings based on their underlying mechanism(s) of action. There is also the possibility of developing specific disease targeted designer probiotics, he added. Elaborating further, he opined that issues related to optimal dosages, time of ingestion, single versus combination formulations, maintenance of viability, and the merits of employing probiotic-derived products would only be answered by undertaking additional research in the field.

Presenting the evidence for their use in adults, Dr. Philip Abraham, Consultant Gastroenterologist, P.D. Hinduja National Hospital, Mumbai, India, stated that probiotics may be useful in preventing antibiotic-associated diarrhoea, including those caused by *Clostridium difficile*. They are also efficacious in reducing the severity and duration of other adult and pediatric diarrhoeas especially those caused by rotavirus. However, their role in prevention of traveler's diarrhoea is equivocal and hence limits their use in this condition.

Dr. Abraham further elaborated on the evidence for their use in chronic and acute conditions specifying that there is increasing evidence indicating that probiotics are an effective adjunct to triple therapy in the eradication of *Helicobacter pylori *infection, exhibiting enhanced eradication rates and reductions in adverse events. He added that although probiotics have shown benefit in improving symptoms of Irritable Bowel Syndrome, current clinical practice guidelines do not recommend their use in treatment regimens. In Inflammatory Bowel Diseases, results are variable although positive benefit has been shown, in preventing relapses in Ulcerative Colitis and Crohn's disease, the most consistent and favourable result being in the prevention and treatment of the pouchitis. Data for the prevention of colon cancer in high-risk individuals however, requires long-term intervention and is therefore hard to come by. There is favourable evidence in animal studies and some evidence in humans, that probiotics can reduce cell proliferation in patients with colonic adenoma [1]. Previous studies suggest that clinical improvement may occur in patients with hepatic encephalopathy due to reduction of fecal urease activity and blood ammonia concentration.

### Why probiotics in diarrhoea in low and middle income countries?

India continues to face disproportionate morbidity and mortality due to diarrhoeal diseases. The 2008 statistics indicate that 18% of the global child diarrhoeal deaths and 77% of diarrhoeal deaths of children in Southeast Asia were from India [3]. Using both culture dependent and culture independent methods for 26 different enteric pathogens, Dr. G.Balakrish Nair, Executive Director of the Translational Health Science and Technology Institute, Gurgaon, Haryana, provided evidence that more than two thirds of the patients with diarrhoea admitted to the Infectious Diseases Hospital in Kolkata excreted more than one enteric pathogen and, some of them excreted as many as six pathogens. Several community-based diarrhoeal surveillance studies conducted in the urban slums of Kolkata, have also shown that enteric pathogens found amongst cases with varied age and socio economic status, matched healthy children sampled as controls thereby substantiating that the extent of exposure to fecal bacteria and enteric pathogens in impoverished settings is huge. This results in tropical enteropathy, increased permeability and modest malabsorption [4]. The consequence of these conditions include impaired nutrient utilization because of altered microbiota, poor oral vaccine uptake, diminished protective efficacy of these vaccines as compared to that in developed countries and requirement of higher oral vaccine dose in the impoverished settings [5]. Available data from developed countries suggest that probiotics are effective against diarrhoea of diverse causes and furnish the evidence for the use of probiotics in prevention and treatment of diarrhoea. In stark contrast, in developing countries, probiotics need to be effective against a complex backdrop of child undernutrition, ingestion of high concentration of fecal bacteria because of poor sanitary conditions, tropical enteropathy, hyperpermeable gut, reduced nutrient absorption and growth faltering. Using this as the background, Dr. Nair opined that probiotics may serve as an ideal intervention strategy to improve gut microbiota and counter enteric pathogens present in such disease endemic areas. Appropriately conducted randomized trials would decide the future significance of probiotics in impoverished settings and would also help to furnish information on the mechanism of action of probiotics in settings quite different from the sanitized West.

Dr. Nair shared the findings of a recent study on 3758 children aged 1-5 years in an urban slum in Kolkata which evaluated the occurrence of first episodes of diarrhoea as primary outcome and composition of fecal microbiota as the secondary outcome. The results of the study revealed that daily intake of a probiotic strain, *Lactobacillus casei *strain Shirota played an important role in prevention of acute diarrhoea in young children in a community setting. These results show a significant protective effect (14%, 95% CI: 4-23%) of the probiotic on acute diarrhea in children during the 24 week period with only 608 children in the Probiotic group (0.88 no. of cases/child/year) developing diarrhoea as compared to 674 children in the Nutrient group (1.029 no. of cases/child/year) during the 24 week study period [6].

Elaborating on the same study Dr. Koji Nomoto, Associate Director, Yakult Central Institute for Microbiological Research, Tokyo, presented details of the variation in the composition of the gut flora in the diarrhoeal samples from the children in the probiotic and nutrient groups. Analysis of the composition was done using Yakult Intestinal Flora - SCAN (YIF- SCAN^®^) which is based on reverse transcription quantitative PCR (RT- qPCR) using specific primers that target bacterialr RNA molecules [7-9]. Dr. Nomoto shared results which revealed that the gut flora was disrupted in the diarrhoeal samples in comparison to the non diarrhoeal samples and how these aberrations in the gut flora could be restored by including a probiotic intervention.

According to Dr. Shinjini Bhatnagar, Professor, Pediatric Biology Centre at the Translational Health Science Technology Institute, Gurgaon, Haryana, at present, there is a lack of sufficient evidence for the use of probiotics in the treatment of acute diarrhoea in the developing world and large randomized controlled studies using specific probiotic regimes in defined groups of children with uniform definitions for clinical outcomes would definitely pave the way forward. She stated that this would help to advance the understanding of specific probiotics in Indian settings where breast feeding rates are higher and microbial colonization of the gut is different. In her opinion, these studies should focus on evaluating possible interactions when probiotics are used as combinations or with other antimicrobials/drugs/zinc.

### Probiotics in mucosal immunology by which mechanisms can probiotics be clinically effective?

As a key physical interface to the external environment, the gut epithelium is a highly dynamic and multi functional barrier. In health, a delicate homeostasis is maintained at intestinal surfaces between the micro organisms present and the host tissues to finely tune responses appropriate to native commensals versus potential pathogens. The protective mucus layer in the intestine controls unchecked bacterial proliferation and also serves as a feedback system to regulate both innate and adaptive immune responses [10]. Dr. S.V. Chiplunkar, Principal Investigator, ACTREC, Tata Memorial Centre, Mumbai discussed the various *in vitro *and *in vivo *studies which have shown that specific strains of probiotics are able to modulate the immune system to protect against infectious diseases and cancer.

She stated that probiotic supplementation leads to a significant increase in phagocytic activity of blood monocytes, granulocytes and augments NK cell activity though a variety of mechanisms. Major gaps, she said, exist in our knowledge about the mechanisms by which probiotics modulate antitumor immunity. Further, different probiotic strains vary in their ability to modulate the immune system and therefore efficacy of each strain needs to be carefully demonstrated through rigorously designed randomized, double-blind, placebo-controlled studies. In conclusion, she emphasized that probiotics play an important role in potentiating innate and acquired immune responsiveness which can be further exploited for modulating anti-cancer immunity [11].

Extending these deliberations further Dr. Masanobu Nanno, Associate Director at the Yakult Central Institute for Microbiological research, Tokyo presented the significant role of *Lactobacillus casei *strain Shirota (LcS) as an important immune modulator that has the ability to prevent recurrence of cancers. This strain of bacteria, he shared, has more than 75 years of research to back its scientific efficacy and has demonstrated a beneficial role in the prevention of recurrence of superficial bladder cancer following transurethral resection [12]. The same strain of bacteria according to him was also effective in preventing the recurrence of colorectal cancer with moderate or severe atypia in subjects who had undergone surgical resection of colon polyps [13]. He elaborated that for understanding the mechanism of action of LcS especially in the prevention of colon cancer, the effect of LcS on colitis associated cancer models was examined where it was observed that administration of LcS prevented the reccurrence of colon cancer by down-regulating IL - 6 production in the colonic mucosa [14]. NK cell activity was also restored in HTLV-I-associated myelopathy patients and the cytotoxic activity increased significantly in healthy subjects who consumed LcS for a period of three weeks. *In vitro *analysis revealed that LcS-induced activation of NK cells is mediated by IL-12 produced by phagocytic cells. Therefore, establishing the fact that LcS plays a significant role in immune modulation which may be the mechanism for the prevention of recurrence of cancers.

Professor Yuichiro Yamashiro from the Probiotics Research Laboratory of Juntendo University Graduate School of Medicine, Tokyo, Japan elaborating two studies of LcS supplementation shared how the administration of LcS, decreased febrile episodes (> 37°C) and the duration of such episodes, reduced constipation frequency and decreased development of diarrhoea thereby bestowing health benefits to elderly people at a Health Service Institution. Data presented by him evidenced that investigation of faecal bacteria disclosed significant increment in numbers of faecal *Bifidobacterium*. Methicillin-resistant *Staphylococcus aureus *and methicillin-resistant coagulase-negative Staphylococci were detected from 1 case and from 5 cases, respectively, out of 42 cases before the clinical study, were not isolated after the administration of LcS [15]. Professor Yamashiro also illustrated that *B. breve *was very effective in the treatment of mucositis (also referred to as mucosal barrier injury), one of the most debilitating side effects of chemotherapy treatment and was also very effective in preventing NEC and infection in preterm infants [16].

### Bacterial vaginosis and probiotics

Dr. P. Hemalatha of the National Institute of Nutrition (NIN), Hyderabad presented information on the use of probiotics in Bacterial Vaginosis (BV), one of the most common vaginal syndrome afflicting premenopausal and pregnant women. Dr. Hemalatha indicated that since reduction of lactobacilli and increase in pH are the main pathogenesis of BV, probiotics are being explored for their role in restoring the vaginal flora. Meta-analysis of several trials with antibiotics have shown a cure rate of 60% but oral lactobacilli treatment followed by antibiotics for treatment of BV have shown more than 80% cure rate after one month. Further, a Cochrane meta-analysis on efficacy of lactobacilli for the treatment of BV showed beneficial outcome with oral probiotics combined with metronidazole or estriol preparation suggesting the need to combine antibiotics with lactobacilli while aiming to treat BV. Sharing findings of a double-blind, randomized, placebo-controlled study conducted by NIN on 67 healthy, non pregnant, married women aged 20 to 40 years from urban slum with BV (Nugents' score > 7) where a vaginal tablet containing at least 10 [9] viable Lactobacilli (*L. brevis, L. salivarius subsp. Salicinius, L. plantarum) *inserted into the vagina daily at bedtime for 8 days demonstrated a cure rate of nearly 80%. Cervico-vaginal lavage (CVL) was collected to measure the concentrations of tumor necrosis factor (TNF)- α, interleukin (IL)-1 β and IL-6 by Quantitative Milliplex ELISA kit. It was interesting to note significant reduction in IL-1β and IL-6 proinflammatory cytokines with lactobacilli treatment, while an increase in IL-6 cytokine was observed with pH adjustment tablet. This is the first study demonstrating the effect of lactobacilli treatment on local proinflammatory cytokines [15].

Merits of this study include control on time of sampling and initiation of treatment, which was during the same phase of the menstrual cycle in all the subjects. Therefore, hormonal influence could be ruled out on the effectiveness of the lactobacilli treatment. Attrition rate was also minimum all through. The major drawbacks of the study include non-use of antibiotics for the treatment of BV and using a pH adjustment tablet instead of a placebo. The vaginal tablet containing three strains of lactobacilli used in the study discussed was well tolerated and no side effects were reported, which was similar to the earlier findings. The strains of lactobacilli used in the study were however, chosen not so much for their ability to adhere and colonize at high levels but primarily because, the preparation when used in Italy showed 100% efficacy. However, as suggested earlier, longer and repeated treatments could be suggested for better out come and must be treated with antibiotics prior to lactobacilli instillation.

### Which is the best carrier matrix for a probiotic?

A question that often comes up in most deliberations is about the choice of the carrier matrix for the delivery of the probiotic strain. Dr. A.K. Srivastava, Director, National Dairy Research Institute, Karnal, India stated that dairy based foods, particularly traditional fermented milks like dahi, yoghurts and dairy beverages which are already an integral part of the traditional Indian diet and are considered functional foods can be explored as the most appropriate carriers of probiotics to optimally express their health promoting functions in the gut. Dairy based probiotic foods represent the largest segment accounting for nearly 65% of the total world functional food market. Presently, dairy based probiotic food industry is firmly placed at the global level completely dominated by Europe, Japan and USA. Although, India is yet to make a mark in the probiotic product development in a big way, major global leaders like Yakult and Nestle, and National player: Mother Dairy have undertaken the formidable task of introducing a new market for probiotics in India having covered much ground in a short span. He emphasized that to launch Indian probiotic products in the country, vital issues such as developing novel indigenous probiotic strains with proven functional efficacy both *in vitro *and *in vivo*, their inter-compatibility, effective dosage and colonization in the human gut, technological parameters during processing of the product, encapsulation and their impact on stability, survival and viability of probiotic strains along with validation of health claims and safety using different animal models as well as in human subjects through clinical studies would need to be worked out. Future success of probiotic formulations, in his opinion, will depend on consumer's acceptance.

Dr. B.S. Ramakrishna, Professor and Head, Department of Medical Gastroenterology, Christian Medical College, Vellore stated that although the FAO/WHO criteria provide excellent general principles for the characterization and evaluation of probiotics in food, they fall short of prescribing the specific tests that may need to be done in an individual case [17]. Given that metagenomic studies and metabolomic profiling of the microbiota and the human host have provided major advances in the ability to characterize the molecular aspects of the intestinal microbiota and understand the human-microbiota interaction, it can be hoped that these will eventually translate to more robust *in vitro *and animal testing procedures that can precede human trials and provide the additional evidence for health benefits. In addition, he emphasized that the health system would need to be harnessed in a way that would allow the reporting and capture of any adverse events that can be potentially related to use of probiotic foods or drugs and provide post-market surveillance of the kind available in the developed world.

Dr. Gianfranco Grompone, Senior Scientist, Danone Research, Institute Pasteur, Montevideo extended the discussion further and illustrated in a very exciting presentation entitled "Reshaping In Vitro Tests for Probiotics: Increasing Predictability by a Multi-Dimensional Approach" how predictability can be improved from in vitro tests for better selection and characterization of potential anti-infectious probiotic strains [1]. Presenting a multi-dimensional integrative approach applied by him and his team to screen 100 LAB strains in vitro, they developed novel host-bacteria heterotypic co-culture systems with Intestinal Epithelial Cells, Peripheral Bone Monocyte Cells and Dendritic cells that were incubated with strains of Lactobacillus, Bifidobacterium or Streptococcus and cytokine secretion profiles determined. In addition mannose adhesion and direct pathogen-inhibition on agar plates were tested. All the results were compiled and analyzed by Principal Component Analysis in order to rank and compare the strains [18]. Two strains displayed distinct activity profiles of great interest for probiotic application, one for anti-inflammatory effect and the other one for anti-infectious purpose. Preliminary results, he shared, show anti-infectious effect *in vivo *using mice infection models.

## Conclusion

It is perhaps most appropriate to conclude with the statement made by Professor Yamashiro who stated that according to World Population Prospects of the UN, we are currently witnessing a transition from high fertility and mortality rates to low birth rate and death. This trend reflects the size and structure of regional populations leading to longevity and shrinking of child population. The results of studies conducted by Professor Yamashiro and his colleagues highlight the role that probiotics will play in the future and the benefits that it will bring for changing structure of world population. Health decision makers have an opportunity to make important strides in the area of probiotic research. A robust pipeline of strain specific benefits that probiotics accrue at a local level is needed to ensure that they are safely, swiftly and successfully delivered to everyone who needs them.

## Competing interests

GBN, PA and NKG are governing body members of the Yakult India Microbiota and Probiotic Science Foundation and declare that they have no competing interest. GBN is the Executive Director of the Translational Health Science and Technology Institute, Gurgaon, Haryana. PA is a Senior Consultant Gastroenterologist and Hepatologist at P.D. Hinduja National Hospital, Mumbai, India. NKG is Distinguished Biotechnology Research Professor, Department of Biotechnology, National Institute of Immunology, New Delhi, India. NH is a Biotechnologist, Secretary and Treasurer of Yakult India Microbiota and Probiotic Science Foundation and the Head of Science, Yakult Danone India Pvt. Limited, a probiotic food company. The report is a reflection of the proceedings of the symposium and not her own views or that of the company.

## Authors' contributions

NH has put together the report on proceedings of the symposium, "Health Impact of Probiotics - Vision and Opportunities." GBN conceptualized the report and gave critical inputs related to the proceedings of the symposium. PA has reviewed the report to verify the factual details of the proceedings of the symposium. NKG was the chairperson of the symposium and critically reviewed the report of the proceedings of the symposium, "Health Impact of Probiotics - Vision and Opportunities". All authors read and approved the final manuscript.

## References

[B1] Gareau MG Sherman PM Walker A Probiotics and the gut microbiota in intestinal health and disease Nat Rev Gastroenterol Hepatol 2010 7 503 514 10.1038/nrgastro.2010.117 20664519 PMC4748966

[B2] Michail S Sherman P Nutrition and Human Health: Probiotics in Pediatric Medicine 2009 Totawa: Humana/Springer Press

[B3] Black RE Cousens S Johnson HL Lawn JE Rudan I Bassani DG Jha P Campbell H Walker CF Cibulskis R Eisele T Liu L Mathers C Global, regional, and national causes of child mortality in 2008: a systematic analysis Lancet 2010 375 1969 87 10.1016/S0140-6736(10)60549-1 20466419

[B4] Humphrey JH Child undernutrition, tropical enteropathy, toilets and handwashing Lancet 2009 874 1032 35 19766883 10.1016/S0140-6736(09)60950-8

[B5] Serazin AC Shackelton LA Wilson C Bhan MK Improving the performance of enteric vaccines in the developing world Nature Immunol 2010 11 769 73 10.1038/ni0910-769 20720580

[B6] Sur D Manna B Niyogi SK Ramamurthy T Palit A Nomoto K Takahashi T Shima T Tsuji H Kurakawa T Takeda Y Nair GB Bhattacharya SK Role of probiotic in preventing acute diarrhoea in children: a community-based, randomized, double-blind placebo-controlled field trial in an urban slum Epidemiol Infect 2011 139 919 926 10.1017/S0950268810001780 20670468

[B7] Kubota H Tsuji H Matsuda K Kurakawa T Asahara T Nomoto K Detection of human intestinal catalase-negative, Gram-positive cocci by rRNA-targeted reverse transcription-PCR Appl Environ Microbiol 2010 76 5440 5451 10.1128/AEM.03132-09 20581195 PMC2918946

[B8] Matsuda K Tsuji H Asahara T Kado Y Nomoto K Sensitive quantitative detection of commensal bacteria by rRNA-targeted reverse transcription-PCR Appl Environ Microbiol 2007 73 32 39 10.1128/AEM.01224-06 17071791 PMC1797142

[B9] Sakaguchi S Saito M Tsuji H Asahara T Takata O Fujimura J Nagata S Nomoto K Shimizu T Bacterial rRNA-targeted reverse transcription-PCR used to identify pathogens responsible for fever with neutropenia J Clin Microbiol 2010 48 1624 1628 10.1128/JCM.01724-09 20351213 PMC2863901

[B10] Borchers AT Selmi C Meyers FJ Keen CL Gershwin ME Probiotics and immunity J Gastroenterol 2009 44 1 26 46 10.1007/s00535-008-2296-0 19159071

[B11] Shida K Nanno M Probiotics and immunology: separating the wheat from the chaff Trends Immunol 2008 29 565 573 10.1016/j.it.2008.07.011 18835747

[B12] Aso Y Akaza H Kotake T Tsukamoto T Imai K Naito S Preventive effect of a *Lactobacillus case *preparation on the recurrence of superficial bladder cancer in a double-blind trial Eur Urol 1995 27 104 109 7744150 10.1159/000475138

[B13] Ishikawa H Akedo I Otani T Suzuki T Nakamura T Takeyama I Ishiguro S Miyaoka E Sobue T Kakizoe T Randomized trial of dietary fiber and *Lactobacillus case *administration for prevention of colorectal tumors Int J Cancer 2005 116 762 767 10.1002/ijc.21115 15828052

[B14] Matsumoto S Hara T Nagaoka M Mike A Mitsuyama K Sako T Yamamoto M Kado S Takada T A component of polysaccharide peptidoglycan complex on *Lactobacillu *induced an improvement of murine model of inflammatory bowel disease and colitis-associated cancer Immunology 2009 128 1 Suppl e170 e180 19740306 10.1111/j.1365-2567.2008.02942.x PMC2753921

[B15] Chiba Y Shida K Nagata S Wada M Bian L Wang C Shimizu T Yamashiro Y Kiyoshima-Shibata J Nanno M Nomoto K Well-controlled proinflammatory cytokine responses of Peyer's patch cells to probiotic Lactobacillus casei Immunology 2009 130 3 352 62 20636824 10.1111/j.1365-2567.2009.03204.x PMC2913215

[B16] Satoh Y Shinohara K Umezaki H Shoji H Satoh H Ohtsuka Y Shiga S Nagata S Shimizu T Yamashiro Y Bifidobacteria prevents NEC and infection in preterm infants Inter J Probio and Prebio 2007 2 149 54

[B17] Joint Food and Agriculture Organization/World Health Organization Guidelines for the evaluation of probiotics in foods 2002 London, Ontario, Canada 1 11

[B18] Ng SC Hart AL Kamm MA Stagg AJ Knight SC Mechanism of action of probiotics: recent advances Inflamm Bowel Dis 2009 15 300 310 10.1002/ibd.20602 18626975

